# Place of Residence Moderates the Risk of Infant Death in Kenya: Evidence from the Most Recent Census 2009

**DOI:** 10.1371/journal.pone.0139545

**Published:** 2015-10-09

**Authors:** Oliver Gruebner, Sven Lautenbach, M. M. H. Khan, Samuel Kipruto, Michael Epprecht, Sandro Galea

**Affiliations:** 1 Department of Epidemiology, Mailman School of Public Health, Columbia University, New York, NY, United States of America; 2 Institute of Geodesy and Geoinformation, University of Bonn, Bonn, Germany; 3 Department of Public Health Medicine, School of Public Health, University of Bielefeld, Bielefeld, Germany; 4 Kenya Bureau of Statistics (KNBS), Nairobi, Kenya; 5 Center for Development and Environment (CDE), University of Bern, Bern, Switzerland; 6 School of Public Health, Boston University, Boston, MA, United States of America; Pennsylvania State University College of Medicine, UNITED STATES

## Abstract

**Background:**

Substantial progress has been made in reducing childhood mortality worldwide from 1990–2015 (Millennium Development Goal, target 4). Achieving target goals on this however remains a challenge in Sub-Saharan Africa. Kenya’s infant mortality rates are higher than the global average and are more pronounced in urban areas as compared to rural areas. Only limited knowledge exists about the differences in individual level risk factors for infant death among rural, non-slum urban, and slum areas in Kenya. Therefore, this paper aims at 1) assess individual and socio-ecological risk factors for infant death in Kenya, and at 2) identify whether living in rural, non-slum urban, or slum areas moderated individual or socio-ecological risk factors for infant death in Kenya.

**Methodology:**

We used a cross-sectional study design based on the most recent Kenya Population and Housing Census of 2009 and extracted the records of all females who had their last child born in 12 months preceding the survey (N = 1,120,960). Multivariable regression analyses were used to identify risk factors that accounted for the risk of dying before the age of one at the individual level in Kenya. Place of residence (rural, non-slum urban, slum) was used as an interaction term to account for moderating effects in individual and socio-ecological risk factors.

**Results:**

Individual characteristics of mothers and children (older age, less previously born children that died, better education, girl infants) and household contexts (better structural quality of housing, improved water and sanitation, married household head) were associated with lower risk for infant death in Kenya. Living in non-slum urban areas was associated with significantly lower infant death as compared to living in rural or slum areas, when all predictors were held at their reference levels. Moreover, place of residence was significantly moderating individual level predictors: As compared to rural areas, living in urban areas was a protective factor for mothers who had previous born children who died, and who were better educated. However, living in urban areas also reduced the health promoting effects of better structural quality of housing (i.e. *poor* or *good* versus *non-durable*). Furthermore, *durable* housing quality in urban areas turned out to be a risk factor for infant death as compared to rural areas. Living in slum areas was also a protective factor for mothers with previous child death, however it also reduced the promoting effects of older ages in mothers.

**Conclusions:**

While urbanization and slum development continues in Kenya, public health interventions should invest in healthy environments that ideally would include improvements to access to safe water and sanitation, better structural quality of housing, and to access to education, health care, and family planning services, especially in urban slums and rural areas. In non-slum urban areas however, health education programs that target healthy diets and promote physical exercise may be an important adjunct to these structural interventions.

## Background

Although substantial progress has been made in reducing childhood mortality worldwide from 1990–2015 (Millennium Development Goal, target 4), achieving target goal 4 remains a challenge in Sub-Saharan Africa [1]. According to the United Nations International Children's Emergency Fund (Unicef) [2], Kenya’s infant mortality rate (IMR), i.e., the probability of dying before the age of one is estimated as 48 per 1,000 live births. This figure is substantially higher than the global average of 34 per 1,000 live births (ibid). In Kenya, IMR is attributed to infectious diseases such as HIV/AIDS, malaria, neonatal sepsis, diarrhea, and acute respiratory infections; to non-infectious diseases and conditions such as birth asphyxia, prematurity and congenital anomalies; as well as to injuries and “other” diseases [3].

Much of this mortality burden can be related to the socio-ecological context into which a child is born [4–6]. To this point, after a rise between the years 1998–2003 [7], Kenya experienced a 7.6% decline in IMR per year between 2003 and 2008, which was achieved through various public health interventions such as the introduction of insecticide treated bednets in malaria endemic regions, HIV/AIDS prevention and treatment, and improvement of water and sanitation facilities [8].

However, Kimani-Murage et al. [9] found that the decline in Kenya’s IMR was taking place significantly slower in urban as compared to rural areas, which might be due to the considerably high urbanization rate of 4% [10] and the development of slums. While urban areas generally provide many advantages for citizens, including improved health and social infrastructure, better income possibilities, or better access to education as compared to rural areas, they are also confronted by a number of serious challenges [11]. Rapidly urbanizing areas, especially those of low-income countries, face limited governance, infrastructural inadequacies, and an inadequate housing sector that often leads to the development of informal settlements including slums [12]. In addition, the urban poor population is limited from accessing health and social services provided in cities because of a lack of financial resources [11]. As a result in low-income countries, child mortality among the urban poor is often higher as compared to urban affluent or rural populations [13, 14].

Health care quality is also typically lower in slums or rural areas of low-income countries as shown e.g. in a study in Bangladesh [15]. In a study of four Kenyan districts, Noor et al. [16] estimated that only 63% of the population would reach a governmental health service facility within one hour. In a nationally representative study, Toda et al. [17] found that only three quarter of all tested health care facilities in Kenya had all family planning or all vaccine commodities in stock and only less than half of all facilities offered delivery services.

In Kenya, a considerably high number of the urban population live in slums [18], which are defined by tenure insecurity, poor structural quality of housing, high population density, and poor access to safe water and sanitation [19]. These contexts increase exposure to disease pathogens that have been found to aggravate infant mortality [5, 20]. Poor livelihood opportunities, limited access to education, health and other social services apply with consequences for child health [21]. Poor maternal education not only in slums further contributes to preventable maternal risk factors such as malnutrition or obesity [4, 22–24].

Yet, there remains much we do not know about socio-ecological risk factors for infant death in Kenya. First, very few studies have investigated rural-urban differences in infant death in Sub-Sahara Africa. Of those studies, many concentrated on all urban versus all rural and did not adjust for inner urban socio-economic differences. Second, individual and socio-ecological risk factors for infant mortality in rural areas may differ from those in urban or slum areas and we are not aware of any study that systematically assessed these differences so far in Kenya.

Therefore, we set out to study infant death in the general population taking stock of the most recent census in Kenya in 2009. Specifically, we aimed at 1) assessing individual and socio-ecological risk factors for the probability of dying before the age of one in Kenya, and at 2) identifying whether living in rural, non-slum urban, or slum areas moderated individual or socio-ecological risk factors for infant death in Kenya.

## Methodology

### Data

We used a cross-sectional study design and based our analyses on the general population in most recent Kenya National Population and Housing Census 2009 [25]. Data was collected by the Kenya National Bureau of Statistics with reference to the night of August, 24th/25th 2009. We followed the guidelines and recommendations to assure Good Epidemiological Practice (GEP) as defined by the German Society for Epidemiology [26]. The study was therefore conducted in accordance with ethical principles and respected human dignity as well as human rights and all information was stored and used anonymously in our analysis. Our study strived to report a qualified risk-communication to the interested public. Household types covered by the census exceeded those considered adequate for this study, since some household types were only covered by a reduced census questionnaire missing important data. We therefore concentrated on housing type 1, “conventional” and excluded “refugees”, “non-conventional” (e.g. schools, hospitals), “institutions”, “travelers”, “vagrants”, and “emigrants”, resulting in a population of 37,919,647. We subsequently concentrated on usual members of the household only, arriving at a population of 35,629,354 living in 8,491,789 households.

### Infant death

Based on 8,491,789 conventional households from the census, we calculated infant death in two steps:

1. Based on questions P32 (month) and P33 (year) "*When was your last child born*?*”*, we identified 1,120,960 mothers that had their last child born between September 1^st^ 2008 and August 24^th^ 2009.
2. Taking the subset produced in 1, infant death was based on the question P36 “*Is this child still alive*?”. Possible options were 2 = No, 3 = One of the Twins, 5 = One of the Multiples, or 6 = Two of the multiples indicating infant death cases in our dichotomized health outcome coded as 1. All other options, such as 1 = Yes, 4 = Both Twins, or 7 = All of the Multiples, and not 9 = DK were coded as 0, indicating that the child was still alive.

Based on this measure, 21,891 (2%) of the mothers’ last-born children (born between September 1^th^ 2008 and August 24^th^ 2009) died before August 24^th^ 2009. It is important to note that our measure does not include mothers that died during pregnancy or delivery and it further does not reflect on those with short birth intervals, which likely underestimates infant mortality and is therefore not directly comparable to infant mortality rates.

### Explanatory variables

We base our study on the conceptual framework for cities and population health of Galea et al. [27] and Gruebner et al. [28] and focus preliminary on differences of living conditions, i.e. individual and socio-ecological risk factors for infant death in rural, urban and slum areas.

#### Demographic variables

For demographic variables, we used individual level information on mothers’ age (range 12–56 years), number of previously born children who died (range 0–14), mothers education (up to primary = 0, secondary+ = 1), and information about child’s sex (girl = 0, boy = 1) including the information whether the child was a twin or multiple (twin/multiple = 2) (Table 1).

**Table 1:** Descriptive statistics for all variables included in the Study, N = 1,120,960.

Variable name	Variable category	Total N	Rural %	Urban %	Slum %
Mother's age*	Up to median (25)	489,132	43.48	42.6	50.68
	25+	631,828	56.52	57.4	49.32
Ever born children alive who died**	Never had a child who died	983,680	86.21	91.22	90.98
	Had at least one child who died	137,280	13.79	8.78	9.02
Mother's education level reached	Up to Primary	1,052,131	96.85	86.05	92.98
	Secondary+	68,829	3.15	13.95	7.02
Child's sex	Girl (ref)	549,540	48.97	49.16	49.12
	Boy	560,236	50.01	49.9	49.94
	Twin or multiple	11,184	1.02	0.94	0.94
Housing quality	Non-durable (ref)	65,725	7.55	2.36	1.1
	Poor	307,765	35.76	9.17	8.64
	Good	455,671	44.1	30.97	42.86
	Durable	291,799	12.59	57.5	47.4
Access to water	Not improved	545,390	57.57	29.3	27.05
	Improved	575,570	42.43	70.7	72.95
Access to sanitation	Not improved	452,874	48.25	22.3	26.53
	Improved	668,086	51.75	77.7	73.47
Household head's sex	Female	313,227	30.06	24.44	17.56
	Male	807,733	69.94	75.56	82.44
Household head's age***	Up to median (34)	545,879	44.93	55.16	66.17
	34+	575,081	55.07	44.84	33.83
Household head's marital status	Not married	104,598	8.97	10.25	9.55
	Married	1,016,362	91.03	89.75	90.45
Residency	Rural	772,100	68.9	/	/
	Urban	289,169	/	25.5	/
	Slum	59,691	/	/	5.3

*Mean age of mothers 26.59 (range 12–56 years, standard deviation [SD]: 6.62).

**Mean number of ever born children that died 0.19 (range: 0–14, SD: .62). Note that this measure excludes infant death occurring within 11 months preceding the census, i.e., the period of the outcome infant mortality.

***Mean age of household heads 37.24 (range: 15–95 years, SD: 13.09).

#### Socio-ecological variables

For capturing the social environment in which a mother was living, we used household level information on household head’s sex (female = 0, male = 1), age (15–95 years) and their marital status (not married = 0, married = 1) (Table 1). For the physical environment, we constructed new variables to account for structural quality of housing, quality of water supply and mode of human waste disposal (sanitation). Quality of housing was constructed from information on material used for floor, wall and roof construction of a household. For the floor of a household, we considered wood, earth and other non-durable materials as minor quality and coded as 0. Cement and tiles were considered durable, coded as 1. Walls made of wood, corrugated iron sheets, grass/reeds, tin and other were considered non-durables and coded 0. Walls made out of stone, brick/block, mud/wood, and mud/cement were considered durable and coded as 1. Main roofing material made of asbestos sheets, grass, tin, mud/dung, and others was considered non-durable and coded as 0. Main roofing material made of concrete, tiles, “Makuti” (i.e., reed/grass type roof finish), or corrugated iron sheets was considered durable and coded as 1. The numbers for each variable were combined and summed up ranging from 0 to 3, with higher values indicating better structural quality of housing (0 = non-durable, 1 = poor, 2 = good, 3 = durable). Following national guidelines for the quality of water access [29], we considered water sources reported as ponds, dams, lakes, stream/river, unprotected spring water, unprotected well, “Jabia”, water vendor, and other as not improved, and coded 0. Protected spring, protected well, borehole, piped into dwelling, piped, and rainwater collection were considered improved water sources and coded 1.

For the type of human waste disposal (sanitation), we considered uncovered latrines, bush and other as unsafe sanitation and coded as 0. Main sewer, septic tank, “Cess pool”, “VIP latrine”, and covered pit latrine were considered as safe sanitation and coded 1.

Socioeconomic status (SES) can be conceptualized in various ways and the most appropriate approach to measure SES depends in part on its relevance to the subject under study [30]. In our study, we conceptualized maternal and household SES based on higher maternal education, better structural quality of housing, improved water, and sanitation considering these variables to have significant relevance to infant death. Other variables of which the majority could also be considered as indicators for SES, such as the type of material used for cooking, type of lighting fuel, a variable indicating whether a household possessed livestock, or the number of ever born children of a mother were excluded to avoid problems with collinearity [31], based on the correlation matrix using a threshold of |r| >.5 to identify high collinearity.

For the place of residence, we constructed a new variable using information on urban status (rural, urban, peri-urban) and residential status (formal, slum), with three categories, i.e., 0 = rural: Households located in rural areas, 1 = non-slum urban: Non-slum urban or peri-urban areas, and 2 = slum: Slums in urban or peri-urban areas. For the ease of interpretation, we solely use the terms *rural*, *urban*, and *slum* in the following although *urban* excludes urban slums but additionally includes non-slum peri-urban areas. Likewise, the term *slum* includes urban and peri-urban slums.

### Analytical methods

First, we fitted bivariate logistic regression models with the binary outcome infant death (1 indicating infant death) in order to identify those predictor variables that were significant at the p<0.1 level, which were used in the subsequent multivariable regression. Second, multivariable logistic regression without interaction terms was used to adjust for all variables considered significant in the first step and to identify the main effects. Third, multivariable logistic regression with interaction terms was used to investigate moderating effects between places of residence, i.e., rural, urban, and slum areas and predictor variables.

We used a backward selection approach to find the most important predictors including interactions based on the lowest AIC values. Further variables were excluded based on epidemiologic reasoning and bivariate model performance. Bivariate and multivariable regression analyses were done with packages MASS [32] and the population attributable fractions were calculated in epiR [33] in the statistical programming language and environment R [34].

## Results

The majority of mothers lived in rural areas (68.9%), followed by mothers living in non-slum urban areas (25.5%) and those that lived in urban or peri-urban slums (5.3%) (cf. Table 1).

### Mothers’ characteristics

The majority of mothers were 25 years or older (for urban 57.4% and rural areas 56.5%, cf. Table 1). In slums however, the proportion of younger mothers was higher (50.7%). The highest proportions of mothers that ever had a child that died (prior to the infant considered in this study) were found in rural areas (13.8%) followed by slum (9%) and urban (8.8%) areas. Furthermore, highest proportions of mothers who had reached a secondary or higher education levels were found in urban areas (14%), followed by slum (7%) and rural areas (3.1%). The sex ratio at birth was slightly higher for boys, showing no association with the place of residence.

### Housing characteristics

Housing characteristics also differed greatly between the places of residence. In rural areas, predominantly *good* structural quality of housing was found (44.1%), whereas *durable* housing structures were found more in both urban (57.5%) and slum (47.4%) areas. More than 70% urban households (both in slums and non-slums) had accessed to improved water, which was only about 40% in rural areas. Similarly, the highest proportion of household with access to improved sanitation was found in urban areas (77.7%), followed by slum (73.5%) and rural areas (51.7%).

### Household heads’ characteristics

The majority of mothers in Kenya had a male household head with highest proportions in slums (82.4%). Most mothers had relatively young household heads except for rural areas where more household heads were 34 years or older (55.1%). The overwhelming majority of household heads were married with only small differences between the different places of residence (ranging from 89.7 in urban areas to 91% in rural areas).

Considering the proportion of infant death by variable category within a place of residence, infant death was most often highest in slums, followed by urban and rural areas (cf. Table 2).

**Table 2:** Infant death by place of residence and each variable category used in this study, N = 1,120,960.

Variable name	Variable category	Kenya		Rural		Urban		Slum	
		Dead	%	Dead	%	Dead	%	Dead	%
Infant	Dead	21,891	2.0	14,462	1.87	5,832	2.02	1,597	2.68
Mother's age	Up to median (25)	10,236	2.09	6,661	1.98	2,735	2.22	840	2.78
	25+	11,655	1.84	7,801	1.79	3,097	1.87	757	2.57
Prev. child death	Never	15,927	1.62	10,066	1.51	4,579	1.74	1,282	2.36
	One or more	5,964	4.34	4,396	4.13	1,253	4.93	315	5.85
Mother's education	Up to Primary	20,773	1.97	14,077	1.88	5,216	2.1	1,480	2.67
	Secondary+	1,118	1.62	385	1.58	616	1.53	117	2.79
Child's sex	Girl (ref)	9,690	1.76	6,454	1.71	2,549	1.79	687	2.34
	Boy	11,247	2.01	7,371	1.91	3,039	2.11	837	2.81
	Twin or multiple	954	8.53	637	8.07	244	8.95	73	12.94
Housing quality	Non-durable (ref)	1,645	2.5	1,465	2.51	158	2.32	22	3.35
	Poor	5,572	1.81	4,940	1.79	506	1.91	126	2.44
	Good	8,616	1.89	6,121	1.8	1,830	2.04	665	2.6
	Durable	6,058	2.08	1,936	1.99	3,338	2.01	784	2.77
Access to water	Not improved	10,939	2.01	8,646	1.95	1,862	2.2	431	2.67
	Improved	10,952	1.9	5,816	1.78	3,970	1.94	1,166	2.68
Access to sanitation	Not improved	9,504	2.1	7,590	2.04	1,432	2.22	482	3.04
	Improved	12,387	1.85	6,872	1.72	4,400	1.96	1,115	2.54
Household head's sex	Female	6,033	1.93	4,353	1.88	1,403	1.99	277	2.64
	Male	15,858	1.96	10,109	1.87	4,429	2.03	1,320	2.68
Household head's age	Up to median (34)	10,923	2	6,450	1.86	3,389	2.12	1,084	2.74
	34+	10,968	1.91	8,012	1.88	2,443	1.88	513	2.54
Household head's marital status	Not married	2,509	2.4	1,602	2.31	727	2.45	180	3.16
	Married	19,382	1.91	12,860	1.83	5,105	1.97	1,417	2.62

Looking at the main effects from the multivariable regression model without interaction (moderation effects) in Table 3, we noted that higher ages (OR 0.978, 0.976–0.980), secondary or higher education (OR 0.862, 95% CI 0.809–0.918), poor (OR 0.684, 95% CI 0.647–0.724), good (OR 0.722, 95% CI 0.683–0.763), and durable (OR 0.833, 95% CI 0.783–0.886) as opposed to non-durable structural quality of housing, improved water (OR 0.945, 95% CI 0.918–0.972) and sanitation (OR 0.903, 95% CI 0.876–0.931) and a married household head (OR 0.796, 95% CI 0.763–0.831) was significantly reducing the risk of infant death.

**Table 3:** Multivariable regression model without and with interaction terms.

	Model 1 without interaction		Model 2 with interaction		
*Focal independent variables*	OR	95% CI (LL-UL)	OR	95% CI (LL-UL)	OR focal variable X moderator
*Individual characteristics of mother*					
Age	0.978	(0.976–0.980)	0.976	(0.974–0.979)	
Number of ever born children alive who died	1.532	(1.512–1.552)	1.552	(1.528–1.576)	
Education level secondary or higher	0.862	(0.809–0.918)	0.934	(0.840–1.036)	
*Individual characteristics of child*					
Child is boy	1.143	(1.112–1.175)	1.143	(1.112–1.175)	
Child is twin or multiple	5.407	(5.039–5.797)	5.408	(5.039–5.797)	
*Housing characteristics*					
Poor structural quality of housing	0.684	(0.647–0.724)	0.665	(0.626–0.706)	
Good structural quality of housing	0.722	(0.683–0.763)	0.690	(0.650–0.733)	
Durable structural quality of housing	0.833	(0.783–0.886)	0.821	(0.764–0.883)	
Improved water	0.945	(0.918–0.972)	0.945	(0.918–0.973)	
Improved sanitation	0.903	(0.876–0.931)	0.904	(0.877–0.932)	
*Individual characteristic of household head*					
Married	0.796	(0.763–0.831)	0.797	(0.764–0.832)	
***Moderator variable***					
Non-slum urban or peri-urban	1.121	(1.082–1.161)	0.833	(0.666–0.996)	
Urban or peri-urban slum	1.487	(1.407–1.570)	1.059	(0.606–1.556)	
***Interaction effects***					
Mother's age X non-slum urban			1.003	(0.998–1.009)	0.980
Mother's age X urban slum			1.011	(1.001–1.021)	0.987
Non-slum urban X nb. of prev. born children died			0.962	(0.933–0.993)	1.494
Urban slum X nb. of prev. born children died			0.943	(0.883–1.004)	1.464
Non-slum urban X mother's education secondary+			0.837	(0.731–0.959)	0.782
Urban slum X mother's education secondary+			1.169	(0.935–1.453)	1.093
Non-slum urban X poor housing			1.243	(1.029–1.509)	0.826
Urban slum X poor housing			1.077	(0.690–1.759)	0.716
Non-slum urban X good housing			1.329	(1.119–1.589)	0.918
Urban slum X good housing			1.133	(0.750–1.806)	0.782
Non-slum urban X durable housing			1.242	(1.044–1.488)	1.020
Urban slum X durable housing			1.029	(0.680–1.642)	0.845
Constant	0.048	(0.044–0.053)	0.051	(0.047–0.057)	
***Model fit***					
AIC	210,477		210,464		
Nagelkerke’s R^2^	0.027		0.027		
Deviance between model 1 and model 2	37.111 (p-value<0.001)				

S.E.: Standard Error, OR: Odds ratio, CI: Confidence intervals, LL: Lower level, UL: Upper level. N = 1,120,960.

In contrast, mothers that had higher numbers of ever born children that died (OR 1.532, 95% CI 1.512–1.552), that had boys (OR 1.143, 95% CI 1.112–1.175), twin or multiples (OR 5.407, 95% CI 5.039–5.797), and mothers that lived in urban non-slum (OR 1.121, 95% CI 1.082–1.161) or slum areas (OR 1.487, 95% CI 1.407–1.570) as opposed to rural areas were at greater risk for infant death.

Population attributable fractions (Table 4) translated into 11.85% (95% CI 9.5–14.14) for mothers below the age of 25 years, 62.73% (95% CI 61.63–63.80) for those who had previous child deaths, 17.73% (95% CI 12.67–22.50) for those with no secondary or higher education, 5.44% (95% CI 2.93–7.89) when the child was a boy, 77.88% (95% CI 76.46–79.22) when it was a twin or multiple birth, 23.34% (95% CI 19.44–27.05) when they lived in a non-durable house, 5.13% (95% CI 2.61–7.59) in a house without improved access to water, 11.65% (95% CI 9.28–13.96) in a house without improved sanitation, and 20.5% (95% CI 17.16–23.70) in a household with an unmarried head. Mothers who were exposed to living in slums versus all other places of residence, had an attributable risk fraction of 28.53% (95% CI 24.84–32.03), while urban mothers as opposed to the other categories had a risk fraction of 4.27% (95% CI 1.39–7.07) and those of rural areas (compared to the other residential areas), a risk fraction of -13.69 (95% CI -16.88–-10.59).

**Table 4:** Population attributable fractions for risk factors.

*Individual characteristics of mother*	Attributable fraction in exposed (%)	95% CI (LL—UL)
Maternal age <25 years	11.85	(9.50–14.14)
Had a child who died	62.73	(61.63–63.80)
No secondary+ education	17.73	(12.67–22.50)
***Individual characteristics of child***		
Child is boy, twin or multiple	17.42	(15.21–19.57)
***Housing characteristics***		
Non-durable structural quality of housing	23.34	(19.44–27.05)
No improved water	5.13	(2.61–7.59)
No improved sanitation	11.65	(9.28–13.96)
***Individual characteristic of household head***		
Not married	20.50	(17.16–23.70)
***Place of residence***		
Rural	-13.69	(-16.88–-10.59)
Non-slum urban or peri-urban	4.27	(1.39–7.07)
Urban or peri-urban slum	28.53	(24.84–32.03)

**Fig 1:**
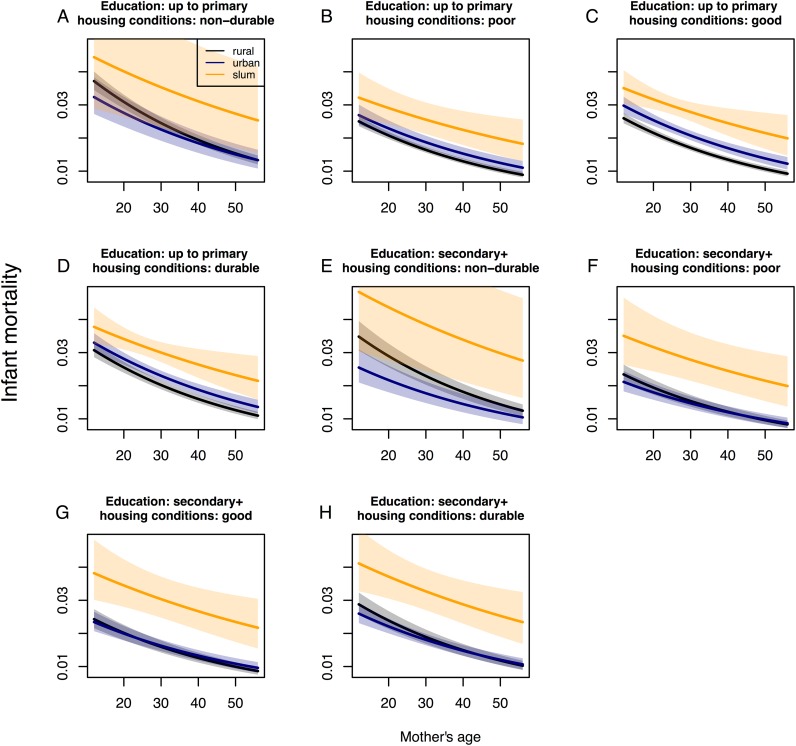
Effect plots for infant death at the individual level for zero previously born children that died. Mother’s age is on the x-axis; infant death is on the y-axis. Confidence intervals at the 95% level are given for the slope of infant death in rural, urban and slum areas. Structural quality of housing is changing from A-D considering mothers education to be primary. Structural quality of housing is changing from E-H considering mothers education to be secondary or higher. Following variables are hold fixed since they were not found to be moderated by place of residence and therefore represent only an offset in the effect plots being held at the reference level: Female infant, unmarried household head, and not-improved water or sanitation.

Taking into account predictors and moderation effects by place of residence in multivariable regression models, we found that in urban areas the likelihood of infant death was significantly lower (OR 0.833, 95% CI 0.666–0.996) compared to other residential areas (cf. Table 3). Furthermore, we found that the effects of mothers’ age, ever born children alive who died, education levels, and structural quality of housing on infant death risk were moderated by the place of residence, i.e. whether mothers lived in rural, urban or slum areas (Table 3, Fig 1). The model with including interactions was significantly better than the model without interactions, indicated by the large delta AIC of 13 as well as by a likelihood ratio test (p-value < 0,001).

Age was moderated by place of residence so that one additional year of age in mothers reduced the risk for infant death by around 2% (OR 0.976, 95% CI 0.974–0.979) in rural areas (Table 3). In urban areas, this effect (i.e. one additional year of age) was 1.003 (95% CI 0.998–1.009) times the magnitude of the effect in rural areas (i.e. 0.980), which translates to a similar 2% decrease of infant death risk by one additional year of age for urban areas, which however was not statistically significant at the 5% level, when all other variables in the model were held at the reference level (i.e., when the mother did not have previously born children that died, had only primary education, and lived in a non-durable house) (Fig 1A), but could be clearly noted in the effect plots across other variable categories, e.g. for those that lived under poor (Fig 1B), good (Fig 1C), or durable housing conditions (Fig 1D). For slum areas, we found that a one-year increase in age was 1.011 (95% CI 1.001–1.021) times the magnitude of the effect in rural areas (0.987). This translates to a 1% decrease in odds for infant death by one additional year of age for mothers that lived in slum areas.

**Fig 2:**
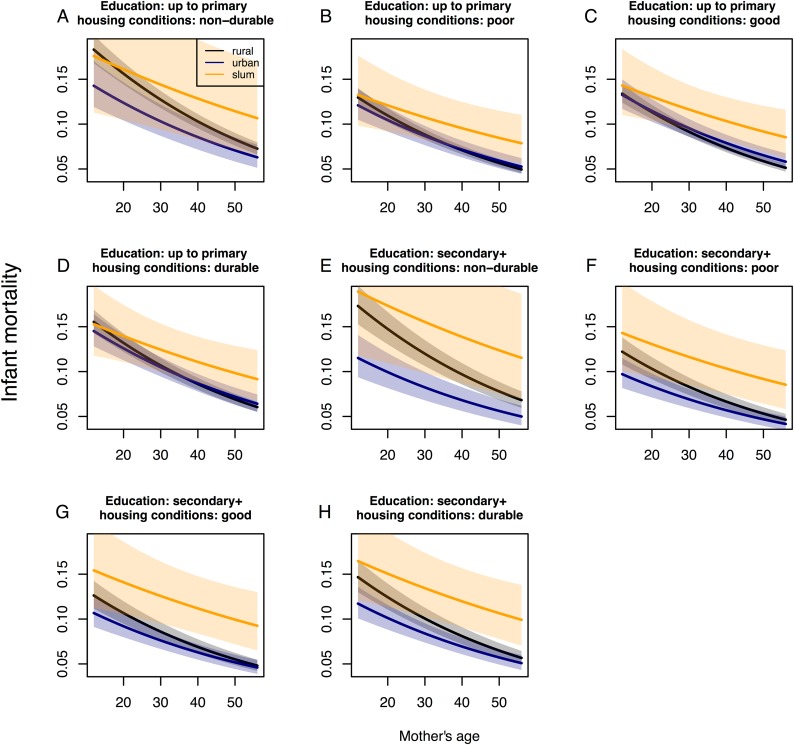
Effect plots for infant death at the individual level for four previously born children that died. Mother’s age is on the x-axis; infant mortality is on the y-axis. Confidence intervals at the 95% level are given for the slope of infant mortality in rural, urban and slum areas. Structural quality of housing is changing from A-D considering mothers education to be primary. Structural quality of housing is changing from E-H considering mothers education to be secondary or higher. Following variables are hold fixed since they were not found to be moderated by place of residence and therefore represent only an offset in the effect plots being held at the reference level: Female infant, unmarried household head, and not-improved water or sanitation.

Any additional child born alive who died prior to those infants considered for the mortality outcome in this study increased the risk of infant death by 55% (OR 1.552, 95% CI 1.528–1.576) in rural areas. For urban areas, this number was 0.962 (95% CI 0.933–0.993) the magnitude of the OR in rural areas (1.494), which translates to a 49% increase in infant death risk by every additional child that died in these areas. For those that lived in slums, the risk for infant death increased by 46% for every additional previous child death, however this relationship was only marginally significant at the 10% level. We identified largest changes in infant death for mothers that did not have previously born children that died conditioned on the reference levels of the other risk factors in the model (Fig 1A) compared to those mothers that had 4 or more previous child deaths and the same reference levels of these risk factors (Fig 2A).

A mother who had reached a secondary education level or higher had around 7% (OR 0.934, 95% CI 0.840–1.036) fewer risk for infant death in rural areas, which however was not statistically significant but still notable in the effect plots by comparing the intercept for rural areas in Fig 1A with the one of Fig 1E. For urban areas, this effect was 0.837 (95% CI 0.731–0.959) times the OR in rural areas (0.782), which translates to a 22% reduction in risk for infant death for better educated mothers in urban areas (Fig 1A and 1E).

Compared to *non-durable* structural quality of housing, *poor* housing in rural areas reduced infant death risk by 33% (OR 0.665, 95% CI 0.626–0.706), *good* housing quality reduced the risk of infant death by 31% (OR 0.690, 95% CI 0.650–0.733), and *durable* housing quality reduced the risk for infant death by 18% (OR 0.821, 95% CI 0.764–0.883). These factors were amplified in urban areas, by 1.243 (95% CI 1.029–1.509) for *poor* housing quality, by 1.329 (95% CI 1.119–1.589) for good housing, and by 1.242 (95% CI 1.044–1.488) for *durable* housing. Thereby they significantly reduced infant death risk in these areas by 17% (in the case of poor housing) and 8% (good housing). However durable housing in urban areas increased infant death risk by 2%, which could also be noted in the effect plots (Fig 1C and 1D).

Child’s sex, access to water and sanitation, and household head’s marital status were not moderated by place of residence and were therefore only expressed as offsets in the effect plots (Figs 1 and 2). Infant death risk was 14% higher in boys (OR 1.143, 95% CI 1.112–1.175) and 441% higher in twins and multiples (OR 5.408, 95% CI 5.039–5.797) as compared to girls. Access to water reduced the risk for infant death by 5% (OR 0.945, 95% CI 0.918–0.973) and sanitation by 10% (OR 0.904, 95% CI 0.877–0.932). Furthermore, mothers that lived under a married household head also had a reduced risk for infant death of about 20% (OR 0.797, 95% CI 0.764–0.832).

## Discussion

We found that younger age of a mother, a higher number of ever born children who died, poor maternal education, child being boy or twin/multiple, living in a non-durable house, without access to improved water or sanitation, and with an unmarried household head were risk factors for infant death in Kenya. Furthermore, we found that these risk factors were moderated by the place of residence and thereby differed in the strength of association across rural, urban and slum areas.

Compared to rural areas, residing in slum or (non-slum) urban areas increased the risk for infant death, when moderating effects were not accounted for. Looking at the moderating effects of place of residence for potentially all socio-ecological factors, we found that living in slum or urban areas decreased the risk for infant death as compared to living in rural areas, conditioned on specific individual level or socio-ecological factors. Living in urban areas was further a protective factor for mothers who had previous born children who died and who were better educated. However, living in urban areas also reduced the health promoting effects of poor and good structural quality of housing. Furthermore, durable housing quality in urban areas turned to be a risk factor for infant death as compared to rural areas. Living in slum areas was also a protective factor for mothers who had previous born children who died, however, it also reduced the promoting effects of older ages in mothers.

Overall, our findings are in line with previous studies that have shown individual and contextual factors related to infant death and that urban-rural and intra-urban differences exist [9, 21, 24]. Therefore, this study did not only confirm existing findings from the literature based on a large population dataset for entire Kenya but also provided novel evidence that infant death and its individual and socio-ecological risk factors could be shaped in part by place of residence. However, according to the census 2009, the lowest portion of mothers (5%) lived in slums, followed by those that lived in urban areas, while the majority of them lived in rural areas. This is in contrast to UN statistics that report more than 50% of the urban population to be slum dwellers [35], which needs to be considered when interpreting our findings.

Older maternal age was a protective factor and was moderated by place of residence. The effect of increasing maternal ages on the risk of infant death showed a close to linear relationship in slums while for rural and urban areas, this relationship was non-linear with particular larger effects at younger maternal ages, i.e. below the age 30 (cf. Fig 1). Since giving birth in adolescence is still very common in Kenya [36], we provided evidence for this phenomenon for rural areas that should be considered for targeted health interventions, especially those that link women’s health and child health as for example the global and national “Every Newborn Action Plan” and movement [37] including young girls’ education and family planning programs [24, 38].

Mothers who had a record of previously born children who died were found at higher risk that their newborn child would also die in all areas, but the risk was particularly amplified in rural areas. In general, this is in line with other studies that found that mothers with a history of child death were at higher risk for infant death [39]. In our study, the risk for infant death increased stronger with the number of previously born children who died in rural and urban areas than in slum areas, leading to an even higher risk of infant death in these areas. High uncertainties for mothers in slums that had a higher number of previously born children that died indicated that we were likely missing some important risk factors for this sub population or that other factors were more important such as, for example, socio-economic barriers to quality health care or health care seeking behavior that might differ in slums as compared to other areas [40]. Furthermore, lower case numbers for mothers living in slums as compared to other areas might also have had an influence on the findings. However, we assume that for the few mothers with higher numbers of previously died infants, individual risk factors outweighed any effect at the place of residence.

The relationship between better maternal education and lower risk for infant death is widely known in the literature [1, 4, 22–24]. Mothers with better education are more likely to have better socioeconomic positions enabling for better nutrition, better housing environment, or better access to health and social care. In our study, an inverse relationship between better education and infant death was significant in urban areas only, though it was also notable in slum and rural areas. There are two explanations for this. First, we could assume that better education was less effective with respect to health knowledge accountable for infant death risk in rural or slum areas. Second, the positive relationships between better education and socioeconomic position, nutrition status, housing environment, or access to health and social care might be very different for different population groups with the largest effects in urban affluent populations as opposed to populations residing in slum or rural areas. Hence, in our study, we could assume that urban areas may provide protective contextual factors that helped elevate the effect of better education for lower risk in infant death. In contrast, comparably poorer socio-ecologic contextual factors in slums or rural areas might have outweighed the effect of better education and should be addressed in these areas [41].

Better structural quality of housing is a known protective factor for health [20, 21, 27, 28] and so decreased the risk for infant death also in our study. However, the health promoting effect of better housing decreased with better housing quality in rural areas with 33%, 31%, and 18% of risk reduction in infant death from non-durable to poor, poor to good, and good to durable housing quality, respectively. The diminishing effect on the inverse relationship between housing quality and infant death was more pronounced in urban areas with only 17% and 8% risk reduction from non-durable to poor and good housing quality, respectively. Durable housing quality in this area however even increased the risk for infant death by 2%. Housing effects health in numerous ways [27, 42] and it can be assumed that one of the links between housing and infant death is through socioeconomic status (SES) that is related to changing lifestyle patterns and maternal obesity, which in turn is a known risk factor for infant death [22]. For example, we could assume that durable materials used for the construction of floor, wall and rooftop indirectly reflected better SES in our study. Research on Kenyan women of higher SES especially in urban areas showed that this group exhibited sedentary lifestyle, and higher consumption of energy, protein, fat, cholesterol, and alcohol were significant predictors of overweight and obesity [43]. Another study by Steyn et al. [44] showed that Kenyan women who belonged to the highest income group, to households where room density was low, electricity or gas was used for cooking, or households had improved water and sanitation were at higher risk for overweight. This was also true in our study, which provided evidence for a likely link between housing quality reflecting better SES and a decreasing promoting effect for infant death that warrants further exploration in subsequent studies.

Independently from place of residence, we found higher risk for infant death in boys, twins, or multiples. While assuming that sons and daughters are treated equally in Sub-Saharan Africa [45], evidence from other studies show that male infants as well as twins or multiples are generally more vulnerable due to biological weakness as compared to female infants or singletons [46, 47]. This was also true in our study, however it could be that other unobserved factors in the preconception environment may also have played a role in the death risk of male and twin/multiple infants [48], so that this relationship should be further investigated in subsequent studies. Better access to water and sanitation is also a known protective factor for health [27, 41] and so decreased the risk for infant death also in our study. Mothers living in a household with a married household head had a significantly reduced risk for infant death as compared to mothers living under a non-married head. We assume that married heads indicated a stable living arrangement that many studies have shown providing a health promoting environment for household members including mother and child [49].

This study had some important limitations. First, a possible recall bias for death records in households might have slightly under estimated our figures. However, since we only used the information on the last-born child, we are confident that this bias is negligible. Second, since we only looked on the last-born child, not all children born alive that died have been considered in this study. Furthermore, our measure did not include maternal mortality [50] that might have led to an underestimation in the risk for infant death in Kenya.

Third, age heaping, the tendency to over report the age of household members particularly by another person such as the household head [51] may have led to under estimation of infant death also in our study. Infants could have been reported older than they actually were and therefore potentially didn’t meet our inclusion criteria for infants in this study i.e., below one year of age. Fourth, the compared population groups, i.e. mothers in rural, non-slum urban, and slum areas varied greatly in size, which should be kept in mind when interpreting our findings. Fifth, there remains large variation unexplained from our study, especially for slum areas. Further studies should apply spatial epidemiological approaches to look into the spatial patterns of model residuals that might help to generate hypotheses and reduce the high uncertainties in slums that were found in our study. Sixth, although we captured the wealth of a household by various explanatory variables such as the structural quality of a household, access to improved water and sanitation, lighting and cooking facilities, and maternal education, other key determinants do exist that are related to socio-economic status (SES) but were not included in this study. These include livelihood opportunities and related employment status especially in slums, access to health and social services including maternal education and childcare [20, 21], practices and attitudes of care and care seeking [52] and malnutrition or obesity [4, 22–24]. Seventh, since our study focused on the general population based on households coded as conventional housing, our study did not cover those mothers that were in the hospital or other institutions during the time of the census night from August 24^th^ to 25^th^. We neither included refugees, emigrants, vagrants, and those who were travelling during that night leading to a further underestimation of infant death in Kenya.

Finally, the combination of exposure factors in our model is only one of several possible combinations and when looking at our findings it should be kept in mind that these are not the only possible risk factors that could predict infant death in Kenya. For example, factors such as the length of preceding birth intervals, size of the baby at birth, duration of breastfeeding, or place of delivery were also found to be important factors for infant death in Kenya [7]. However, this information was not available from the census and hence could not be used in our study.

Notwithstanding these limitations, this is the first study to our knowledge that used a complete national census to investigate risk factors for infant death at the individual level in Kenya. We showed that individual and socio-ecological risk factors for infant death differed for rural, non-slum urban, and slum areas, indicating an advantage (reduced risk for infant death) for urban and rural over slum residents. While urbanization and slum development continues in Kenya, public health interventions should invest in healthy environments that ideally would include improvements to access to safe water and sanitation, better structural quality of housing, and to access to education, health care, and family planning services, especially in urban slums and rural areas. In non-slum urban areas however, health education programs that target healthy diets and promote physical exercise could be fruitful.
